# Yttrium-90 Transarterial Radioembolization as a Bridging Therapy to Liver Transplant in a Toddler With Pretreatment Extent (PRETEXT) IV Hepatoblastoma

**DOI:** 10.7759/cureus.87107

**Published:** 2025-07-01

**Authors:** Justin Rehder, Jay Desai, Tyric Goode, William Rudder, Jennifer Fox, Kathleen Anderson, Alexandra Wilder, Vidyaratna Fleetwood, Jerome Kao, Kirubahara Vaheesan, Chintalapati R Varma, Ajay Jain, Mustafa Nazzal

**Affiliations:** 1 Neurology/Surgery, Saint Louis University School of Medicine, Saint Louis, USA; 2 Surgery, Sisters of St. Mary (SSM) Health Saint Louis University Hospital, Saint Louis University School of Medicine, Saint Louis, USA; 3 Surgery, Saint Louis University School of Medicine, Saint Louis, USA; 4 Surgery/Transplant Surgery, Sisters of St. Mary (SSM) Health Saint Louis University Hospital, Saint Louis University School of Medicine, Saint Louis, USA; 5 Interventional Radiology, Sisters of St. Mary (SSM) Health Saint Louis University Hospital, Saint Louis University School of Medicine, Saint Louis, USA; 6 Pediatric Gastroenterology, Sisters of St. Mary (SSM) Health Cardinal Glennon Children's Hospital, Saint Louis University School of Medicine, Saint Louis, USA

**Keywords:** bridging therapy, hepatoblastoma, liver transplatation, posttext, pretext, surgical case reports, transarterial radioembolization, y90 sirt

## Abstract

Hepatoblastoma (HB) is a rare but highly malignant tumor that commonly arises in utero. The mainstay treatment is surgical resection, with adjuvant treatment options including chemotherapy, hepatic artery chemoembolization, and liver transplantation. Yttrium-90 (Y90) transarterial radioembolization (TARE Y90) is a developing treatment option that utilizes radioactive beads delivered specifically to blood vessels supplying the tumor. This treatment method has previously been utilized in the treatment of adult tumors but has rarely been utilized in the pediatric population.

In this report, we discuss the case of a toddler with an unresectable HB who underwent TARE Y90 as an adjuvant treatment to chemotherapy, ending in a successful liver transplantation. This patient was a 19-month-old female who presented with an abdominal mass and laboratory findings significant for anemia, thrombocytosis, elevated lactate dehydrogenase (LDH), direct hyperbilirubinemia, and alpha-fetoprotein (AFP) elevation. A computed tomography (CT) scan and magnetic resonance imaging (MRI) displayed a large, multilobular mass involving all lobes of the liver without evidence of metastasis, and biopsy results were consistent with HB. Following four rounds of chemotherapy, an MRI of the abdomen revealed a decrease in tumor size. Due to the continued uptrend of AFP raising concern for lack of control of the tumor, treatment with TARE Y90 microspheres radioembolization as a bridging therapy was initiated. Repeat scans showed an interval increase in the HB size, likely secondary to tumor necrosis with edema and hyperemia. Nine months following the diagnosis, the patient underwent an orthotopic liver transplant and a sixth cycle of chemotherapy.

This report illustrates the use of TARE Y90 in a pediatric patient diagnosed with HB, thereby inducing a direct cytotoxic effect on tumor cells. Adjunctive therapy Y90 radioembolization worked by controlling tumor size and AFP levels, facilitating the crucial aspect of liver transplantation with tumor resection with negative margins. The patient did not suffer any of the known side effects of TARE Y90 therapy, which include, but are not limited to, post-embolization syndrome, biliary complications, stricture, cholangitis, pulmonary complications, and gastrointestinal ulcers. This therapy, along with chemotherapy, has the potential to be a novel approach as a bridge to resection or orthotopic liver transplant in HB patients. Further case reports and a manuscript compiling the use of Y90 radioembolization therapy in pediatric HB patients would be useful in evaluating the efficacy of the treatment in pediatric patients.

## Introduction

Hepatoblastoma (HB) is a highly malignant tumor that arises from primitive hepatic stem cells. HB historically presents as an asymptomatic abdominal mass that sometimes occurs in conjunction with various symptoms, including fever, fatigue, anorexia, and weight loss. While rare, more severe symptoms of HB include rupture of the tumor and obstructive jaundice [1]. Although HB is an extremely rare condition with an estimated 100 pediatric cases diagnosed in the United States each year, it is one of the most common causes of malignant liver tumors in children and has been increasing in prevalence. It is hypothesized that HB arises in utero, as the highest incidence has been observed in infants at birth [2]. 

The mainstay of definitive treatment for HB remains surgical tumor resection or liver transplantation. Due to the five-year survival rate of HB being relatively low (74%) compared to other childhood cancers [2], the exploration and use of novel bridging treatments is integral. Such treatment options include, but are not limited to, chemotherapy, hepatic artery chemoembolization, and radioembolization with yttrium-90 (Y90) microspheres [3]. One such technique, Y90 transarterial radioembolization (TARE) therapy, commonly referred to as TARE Y90 therapy, has recently emerged as an effective bridging option for HB, specifically in patients who are initially unable to receive resection of tumor cells through surgery. Y90 therapy utilizes radioactive beads that are delivered to the blood vessels supplying the tumor via a catheter [4]. The precision of these micron-sized particles is what enables therapeutic results in patients who are ineligible for or already receiving more conventional treatments. Treatment with Y90 also allows for a significantly extended median time-to-progression of carcinoma compared to traditional transarterial chemoembolization [5]. 

Although Y90 therapy has been previously used in adult patients, Y90 has recently been utilized in pediatric patients with HB due to its ability to minimize damage to healthy liver tissue while simultaneously downstaging cancerous tissue to allow for surgical removal [4]. Minimal data exists concerning the use of TARE Y90 in pediatric treatment of refractory or non-responsive liver tumors. In our study, we report a three-year-old female pediatric patient with HB who underwent Y90 radiation particle treatment, leading to acquisition of eligibility for a liver transplant.

## Case presentation

A 19-month-old female patient with no significant past medical history presented to the office with a one-week history of increasing irritability and a one-day history of fever. During this time, her stools became more solid and pasty in color. The physical examination was significant for fussiness and abdominal distention with pain on palpation. A kidney, ureter, and bladder (KUB) X-ray study showed peripheral displacement of large bowel loops, relative paucity of small bowel gas, and calcific densities over the right mid-abdomen. Abdominal ultrasonography revealed a large heterogeneous mass with internal calcification arising from the liver, distorting adjacent anatomy. 

**Figure 1:**
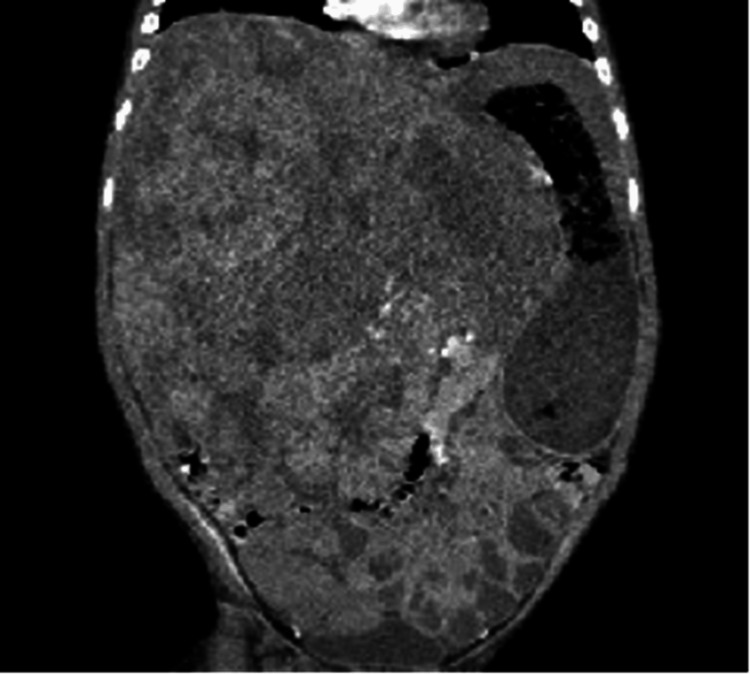
Original non-contrast CT scan demonstrating mass effect of the patient’s hepatoblastoma. CT: computed tomography

The patient was referred to our tertiary care hospital with findings of abdominal mass, anemia, thrombocytosis, and elevated lactate dehydrogenase (LDH). Hepatic function panel was notable only for mild direct hyperbilirubinemia. A computed tomography (CT) scan of the abdomen revealed a large heterogeneous hepatic mass involving all segments (Pretreatment Extent (PRETEXT) Stage IV), with mass effect on multiple structures, including the portal vein and hepatic artery (Figure 1). Magnetic resonance imaging (MRI) was performed to further characterize the mass, which revealed a very large, multilobular mass arising from the right lobe of the liver and involving all lobes of the liver with no evidence of metastasis. The levels of blood tumor marker alpha-fetoprotein (AFP) were markedly elevated to 36,971 ng/mL. 

The patient underwent liver biopsy, which showed results consistent with HB. Tumor treatment was initiated with a chemotherapy regimen (AHEP1531 Group C C5VD protocol) from a Children's Oncology Group (COG) clinical trial being implemented at our institution. This regimen consisted of six cycles of cisplatin (CDDP), 5-fluorouracil, vincristine, and doxorubicin (C5VD). The patient tolerated the first three cycles of chemotherapy well. MRI of the abdomen two months following the diagnosis revealed decreased size of the hepatic mass from 10.4 x 15.3 x 18.5 cm to 10.3 x 12.6 x 15.9 cm, but still appeared to involve all hepatic sections. A month later, the patient tolerated the fourth cycle of chemotherapy well, and follow-up CT showed further reduction of the mass to 10 x 10.9 x 13.7 cm. Almost three months following the diagnosis of HB, the patient was registered as a Status 2B on the United Network for Organ Sharing (UNOS) Patient Waiting List. 

**Figure 2:**
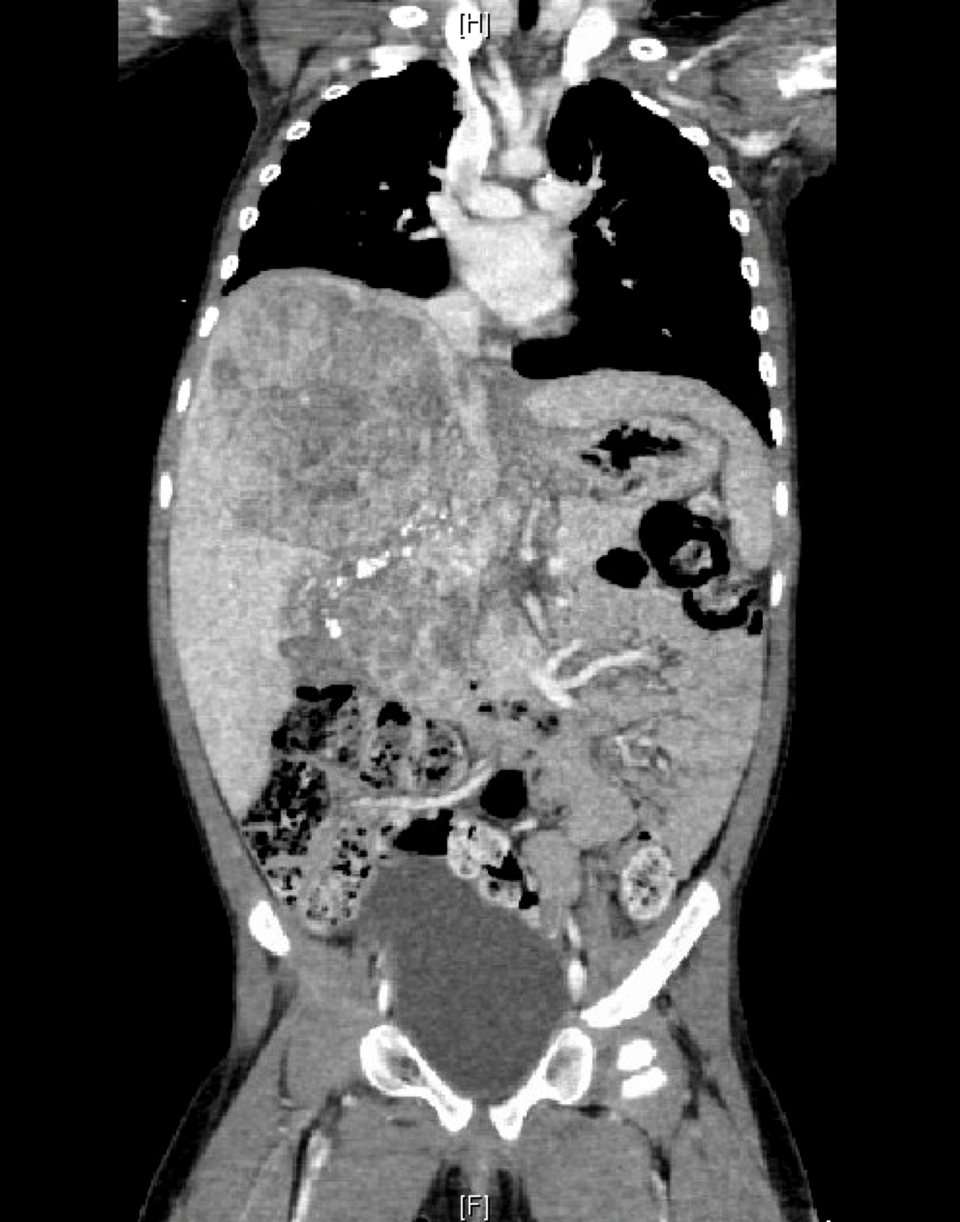
First CT scan two weeks following TARE Y90 treatment demonstrating decreased mass effect of the hepatoblastoma. CT: computed tomography; Y90: yttrium-90; TARE: transarterial radioembolization

However, the patient’s AFP marker continued to trend up to 55,246 ng/mL (previously 41,587 ng/mL). Without evidence of metastatic disease on imaging, there was concern about the lack of control of the tumor. Thus, a decision was made to begin Y90 radioembolization treatment as a bridging therapy. Pre-Y90 mapping revealed the tumor had three blood supplies: ~70% from the replaced right hepatic artery, ~25% from the right hepatic artery, and <5% from the phrenic artery. The patient received 59.32 mCi TARE Y90 microspheres (selective internal radiation (SIR)-spheres) via the hepatic artery without any side effects. Subsequent CT scans immediately post-radioembolization revealed a decrease in mass size to 10.4 x 8.2 x 12.4 cm (Figure 2). Repeat scans a month later revealed an interval increased size of the HB, likely secondary to an interval increase of tumor necrosis, with the extent of the mass similar to prior scans and continued evidence of no metastases. 

**Figure 3:**
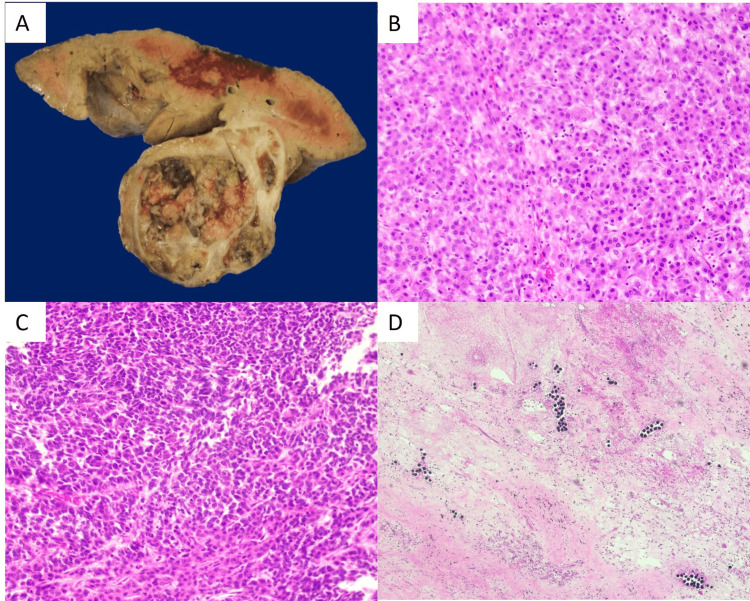
Pathology findings: (A) Gross cross-section of the hepatectomy specimen revealing a large, partially necrotic, and hemorrhagic mass involving the right liver lobe. (B, C) Microscopic examination revealing areas of viable hepatoblastoma with both fetal (B) and embryonal patterns (C) (H&E, 200X). (D) Tumor necrotic areas containing chemoembolization beads are seen throughout the tumor. H&E: hematoxylin and eosin

**Figure 4:**
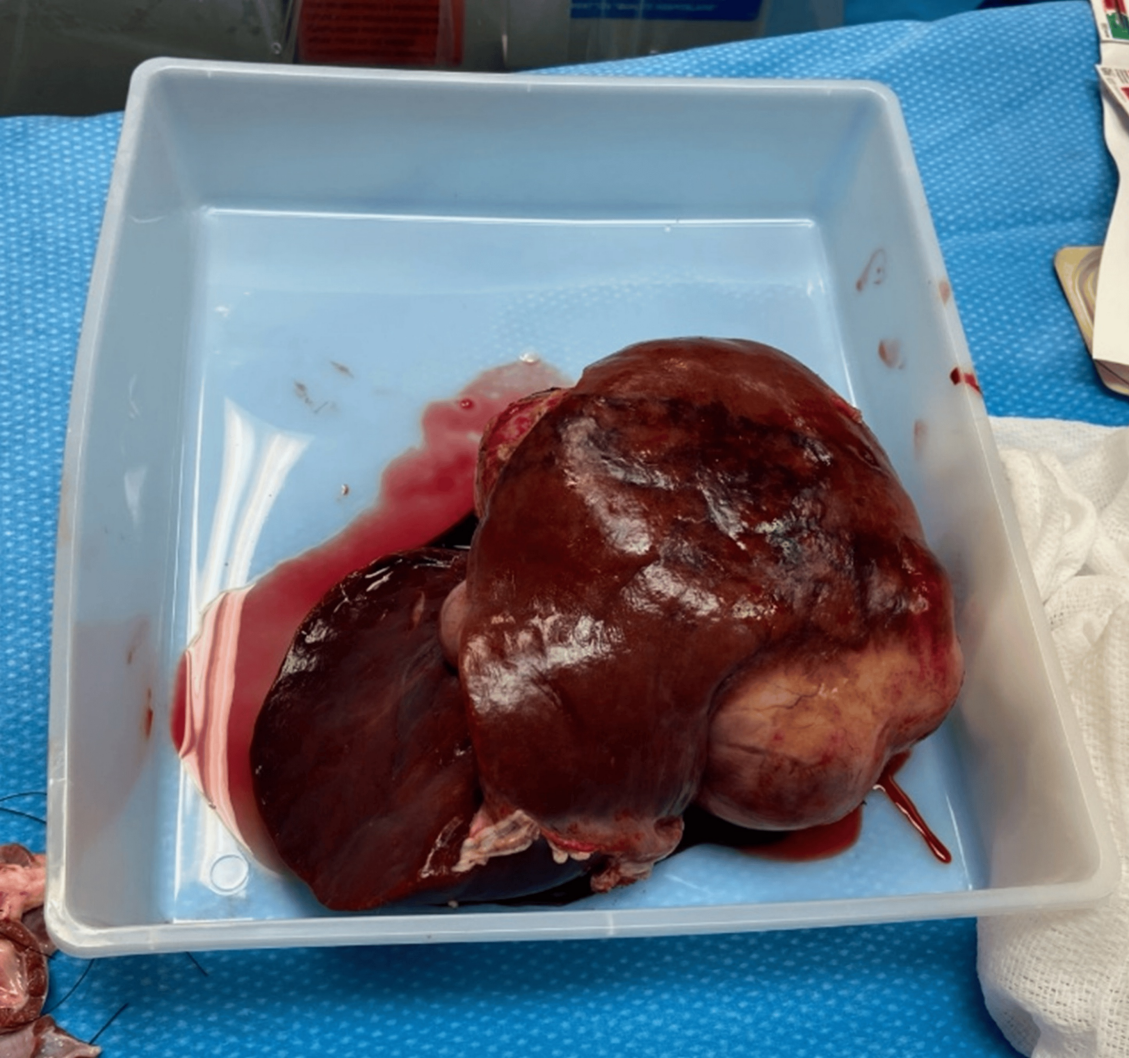
Gross image of the explanted liver.

The patient received her fifth cycle of chemotherapy and had a hospitalization for neutropenic fevers soon after. A routine CT scan did not reveal any significant changes in the tumor size, with AFP levels at 66,419 ng/mL, elevated from 34,457 ng/mL one month prior. This CT demonstrated a Post-treatment Extent (POSTTEXT) Stage III HB. Nine months following her diagnosis, an ABO compatible liver full liver graft became available from a donor of compatible size and age, and the patient underwent orthotopic liver transplant utilizing bicaval implantation technique. Of note, during the hepatectomy, the recipient liver appeared to have dense adhesions to the diaphragm. The portal vein of the donor was anastomosed to the recipient portal vein in an uninterrupted fashion, and so was the common hepatic artery of the donor to the proper hepatic artery of the recipient. Choledocho-choledochostomy was performed for the biliary anastomosis. Intraoperative ultrasound confirmed good arterial and venous waveforms. Induction immune prophylaxis used pulse steroids, and maintenance immunosuppression was with triple immunosuppression including tacrolimus, mycophenolate, and steroids. Tissue pathology of the explanted liver demonstrated multifocal HB, mixed epithelial and mesenchymal type, with teratoid components (Figure 3). All resection margins were negative for tumor (Figure 4). The patient recovered well postoperatively, was discharged home, and two months later received her sixth cycle of chemotherapy and tolerated it well. At the time of publishing, the patient is two years status post-liver transplantation and is doing well without any complications. Monitoring protocols include CT abdominopelvic scans and AFP levels, which have both been stable.

## Discussion

Liver tumors are not common in children. HB is a rare malignancy that arises from the liver stem cell line. HB has been associated with various risk factors, including prematurity [6], pre-eclampsia and poly/oligohydramnios, as well as high pre-pregnancy weight and infertility treatments in the gestational mother [7]. Most notably, very low birth weight has been consistently found to be a risk factor for the development of HB [8]. Over the years, the survival rate has significantly improved with the advancement of chemotherapeutic agents, surgical techniques, and the successful outcomes of liver transplantation. Surgical removal or liver transplantation is always the goal for curative intent [8]. 

TARE Y90 sends microradiation particles directly into the blood vessels that feed the tumor. It induces a direct cytotoxic effect on these tumor cells. Although this treatment is not curative, it aims at locoregional control, expansion, and downstaging the tumor. TARE Y90 has been shown to be superior to chemoembolization in terms of time to disease progression, toxicity, and quality of life in adults with hepatocellular carcinoma (HCC) [9]. It has also been utilized as a bridging locoregional therapy for HCC patients awaiting liver transplant. Its use in HB patients has recently been proposed through anecdotal cases, especially in children, where the tumor is refractory to chemotherapy. 

Our patient was diagnosed with an HB tumor staged PRETEXT IV, involving all four segments of the liver [10]. PRETEXT HB classification is defined as: Stage I, involving one segment of the liver; Stage II, involving two non-adjacent segments; Stage III, involving three non-adjacent liver segments or two adjoining segments; and Stage IV (as in this case), where all four sections are involved [11]. This categorization, as well as the unresectable nature of the tumor, automatically classifies this patient’s HB as high-risk [1]. MRI was the main imaging modality used for classifying the tumor, and liver biopsy was used to definitively diagnose the tumor. The patient began treatment being studied in the ongoing clinical trial by COG. This regimen intensified the C5V (cisplatin (CDDP), 5-fluorouracil, vincristine) regimen with high-dose CDDP and the addition of doxorubicin (C5VD) [1]. Within this chemotherapy regimen, cisplatin is the most commonly used agent as it can provide curative treatment among low-risk HBs. Doxorubicin has also shown effectiveness, though it carries a significant risk of side effects in myelosuppression and cardiotoxicity [12]. Four cycles of chemotherapy resulted in reduced tumor size, yet at the end of the therapy, the tumor still took up an area of 1,493.3 cm³ in the patient’s abdominal cavity. This was expected to create significant technical difficulties and a struggle with liver transplantation.

The treatment itself had no complications in our patient. The patient did not suffer any of the known side effects of TARE Y90 therapy, which include, but are not limited to, post-embolization syndrome, biliary complications, stricture, cholangitis, pulmonary complications, and gastrointestinal ulcers. Following treatment, the patient's HB continued to display a POSTTEXT III classification. POSTTEXT HB staging also includes four different classifications. This is used following a given treatment in order to re-evaluate the treatment strategy/necessity. Classification is defined as: Stage I, tumor is completely resected; Stage II, microscopic residual tumor remains after resection; Stage III, no distant metastases and at least one is true - (i) the tumor is unresectable/gross residual tumor remains, or (ii) there are positive extrahepatic lymph nodes; and Stage IV, distant metastases are involved regardless of the extent of liver involvement [11]. The Y90 therapy worked in regressing the tumor size from previous pre-Y90 therapy CT scans to a volume of 1,057.5 cm³. Later imaging demonstrated an increase in the mass size, yet this was attributed to tumor necrosis leading to edema and hyperemia. Despite the increase in AFP levels, subsequent scans continued to display a stable tumor size, and besides a hospitalization for chemotherapy-related neutropenic fevers, the patient reported no long-term adverse effects. Intraoperatively, although there were some adhesions of the liver to the diaphragm, there was no apparent radiation injury to other structures, including the bile duct or the hepatic artery.

Further investigation revealed an additional case series published by Balli et al. [12], wherein TARE Y90 was used as an adjuvant treatment in three toddlers diagnosed with HB. One patient had a low-risk HB in which Y90 treatment aided in completely eradicating the tumor tissue. The other two patients (similar to our case) demonstrated significant tumor size reduction, with one toddler undergoing left hepatectomy and the other awaiting liver transplantation. The two patients had Y90 chosen as an adjuvant treatment due to a lack of significant response to chemotherapy. Another had Y90 added due to doxorubicin-induced cardiotoxicity. No patient in the case series demonstrated any side effects attributable to TARE Y90 treatment [12].

## Conclusions

Overall, TARE Y90 therapy was a successful adjunctive treatment in this pediatric patient with impressive HB size. This therapy, along with chemotherapy, has the potential to be a novel approach as a bridge to resection or orthotopic liver transplant in HB patients. On a broader scale, further case reports and a manuscript compiling the use of Y90 radioembolization therapy in pediatric patients with radiosensitive tumors would be useful in evaluating the efficacy of the treatment in pediatric patients.
